# Novel Hybrid Catalysts of Cysteine Proteases Enhanced by Chitosan and Carboxymethyl Chitosan Micro- and Nanoparticles

**DOI:** 10.3390/polym16223111

**Published:** 2024-11-06

**Authors:** Marina Holyavka, Yulia Redko, Svetlana Goncharova, Maria Lavlinskaya, Andrey Sorokin, Maxim Kondratyev, Valery Artyukhov

**Affiliations:** 1 Biophysics and Biotechnology Department, Voronezh State University, 1 Universitetskaya Square, 394018 Voronezh, Russia; redkoju@yandex.ru (Y.R.); olshannikovas@gmail.com (S.G.); maria.lavlinskaya@gmail.com (M.L.); andrew.v.sorokin@gmail.com (A.S.); ma-ko@bk.ru (M.K.); artyukhov@bio.vsu.ru (V.A.); 2 Laboratory of Structure and Dynamics of Biomolecular Systems, Institute of Cell Biophysics of the RAS, 3 Institutskaya Street, 142290 Pushchino, Russia

**Keywords:** ficin, papain, bromelain, chitosan, carboxymethyl chitosan, complexation, microparticles, nanoparticles

## Abstract

Micro- and nanoparticles of chitosan and carboxymethyl chitosan were synthesized, both with and without ascorbic acid. Methods were developed to form complexes between these micro- and nanoparticles and plant proteases—ficin, papain, and bromelain. It was demonstrated that the activity of cysteine protease complexes with carboxymethyl chitosan micro- and nanoparticles was higher compared to those with chitosan micro- and nanoparticles. Additionally, the complexes of ficin, papain, and bromelain with chitosan and carboxymethyl chitosan micro- and nanoparticles synthesized in the presence of ascorbic acid exhibited greater proteolytic activity than those formed with particles prepared without ascorbic acid. Molecular docking studies revealed that the amino acid residues of ficin, papain, and bromelain primarily interact with chitosan and carboxymethyl chitosan through hydrogen bonding and hydrophobic interactions. The amino acid residues in the active sites of these enzymes participate in a complex formation, which likely contributes to the increased activity and stability of cysteine proteases in complexes with chitosan and carboxymethyl chitosan micro- and nanoparticles.

## 1. Introduction

Cysteine proteases of plant origin play a significant role in medicine, the pharmaceutical industry, and cosmetology, owing to their extensive use in traditional medicine, particularly in India and other southern regions [1]. Among these enzymes, papain (EC 3.4.22.2) from *Carica papaya* is the most extensively studied cysteine protease. Papain exhibits endopeptidase, amidase, and esterase activities [2]. The latex of *Carica papaya* has traditionally been used to treat warts, corns, and cancer; the roots have been utilized for piles and yaws; the leaves have been used for alleviating nervous pain; and the fruit has been used for treating infected wounds, and malignant tumors [3]. Papain is widely used in wound debridement to remove necrotic tissue from chronic wounds and burns [4]. Ficin (EC 3.4.22.3), the primary proteolytic enzyme in *Ficus* latex, shares similar specificity with papain. Ficus has been employed in medicine for the treatment of ulcers, complications related to bile secretion, psoriasis, anemia, piles, jaundice, nasal and oral hemorrhages, and blood disorders, as well as serving as an antidysenteric, purgative, and emetic agent. Additionally, it has been used to treat nematode infections and promote wound healing [5]. Bromelain (EC 3.4.22.4) is an endopeptidase derived from Ananas comosus and is recommended for the treatment of burn wounds. It is a key component of the drug NexoBrid^®^, MediWound, Yavne, Israel (known as Debrase in the USA) [6]. Due to its low toxicity, bromelain is an effective agent for combating chronic inflammatory diseases. It exhibits immunomodulatory activity and accelerates tissue repair by depolymerizing intercellular structures and altering vascular permeability [7]. Additionally, bromelain is used in cosmetology for the treatment of acne [8] and possesses anti-biofilm activity [9].

Various strategies can be employed to enhance enzyme stability and optimize their kinetic properties. Metagenomics provides access to the full spectrum of biodiversity, allowing for the exploration of both ancient and contemporary enzymes [10,11]. Direct evolution enables the enhancement of specific enzyme features under desired conditions, effectively mimicking natural selection [12]. Site-directed mutagenesis allows for the refinement of the active site [13] or even the creation of new enzymatic activities [14]. Immobilization, a technique developed to facilitate enzyme recovery and reuse—particularly for costly biocatalysts—also streamlines downstream processing, supports diverse reactor configurations, and ensures a stricter control of reactions, thereby preventing product contamination by the enzyme [15]. The interaction between the enzyme and the support surface during immobilization can lead to structural distortions [16,17], often resulting in new enzyme forms with altered catalytic properties, which may be less effective than the native enzyme. However, by employing various immobilization strategies and techniques, it is possible to achieve immobilized enzymes with improved selectivity, specificity, or activity for a given process [18]. Enzyme inhibition can also be mitigated through various approaches [19]. Importantly, enzyme immobilization is compatible with other techniques designed to enhance enzyme properties [20,21,22]. Therefore, immobilization is not merely a method for enzyme recovery; it can be a crucial step in developing a biocatalyst with optimal characteristics [23]. Like any other enzyme, proteases can benefit from immobilization to enhance their applications. However, proteases possess certain unique characteristics that can complicate the effects of immobilization. When used with small substrates, such as in peptide synthesis or a racemic mixture resolution [24], proteases behave similarly to other enzymes. However, when utilized as proteolytic agents, the substrate tends to be large and relatively rigid—sometimes even larger than the enzyme itself. This presents challenges when using porous support as it may limit the effectiveness of proteases immobilized within these pores. Therefore, the pores of the support material must be large enough to accommodate not only the enzyme but also the substrate [23,25,26].

Immobilizing proteases on porous supports can yield enzymes with a proper orientation and preserved structure; however, this may also lead to reduced activity in the intended reaction. This reduction in activity occurs because only a small fraction of enzyme molecules located on the outer surface of the support remains accessible to large substrates [21,25,26]. Another distinctive feature of proteases is their tendency to undergo autolysis [27], meaning they can degrade themselves by attacking other protease molecules, as they too are proteins. Beyond enabling enzyme recovery, immobilization offers additional benefits, such as enhanced enzyme stability [28,29]. When carefully designed, immobilization can also be combined with enzyme purification [30]. Consequently, immobilization technology is regarded as a powerful tool for improving enzyme properties through the use of solid support materials [31]. In recent years, biotechnology has introduced a variety of micro- and nanoscale supports for enzyme immobilization [32]. These micro- and nanoscale supports provide a large surface area for efficient enzyme loading while minimizing resistance to substrate mass transfer [33], making them an effective approach to enhancing enzyme performance. The exceptional properties of micro- and nanoparticles have led to numerous applications, both in industries such as in bioremediation and catalysis and in biomedical fields, particularly in drug delivery systems [34].

Chitosan, one of the most abundant natural biopolymers, is derived from chitin. It is highly valued for its exceptional biological, physicochemical, and antimicrobial properties, as well as its non-toxicity, making it an excellent eco-friendly material. Due to these unique and remarkable characteristics, chitosan micro- and nanoparticles are promising candidates for a wide range of applications, including wastewater treatment, biosensing, diagnostics, pharmaceuticals, tissue engineering, enzyme immobilization, and food packaging. Consequently, the aforementioned studies suggest that chitin-derived supports used for immobilizing various industrial and therapeutic enzymes provide an excellent platform for achieving high-yield bioprocesses [35,36].

Carboxymethyl chitosan is a carboxylated derivative of natural chitosan (Figure S1). Its main advantage lies in its ampholytic nature, attributed to the presence of both positively charged amino groups and negatively charged carboxylic groups within a single macromolecule. As a result, carboxymethyl chitosan can interact with both cations and anions, as well as with other compounds capable of forming hydrogen bonds with the hydroxyl groups of glucopyranose rings. Additionally, as a derivative of non-toxic, non-immunogenic, and biocompatible chitosan, carboxymethyl chitosan retains these valuable characteristics, making it a promising candidate for biomedical applications [37]. Various carboxymethyl chitosan-based drug delivery systems have been developed for the targeted transport of antibiotics [38], cytostatics [39], and other therapeutic substances [40]. Furthermore, carboxymethyl chitosan is highly effective for wound dressing production due to its gel-forming properties and its ability to maintain a moist environment while removing exudate [41].

Micro- and nano-catalysts offer a promising approach by combining the advantages of both homogeneous and heterogeneous catalysis [42]. These catalysts enable rapid and selective chemical transformations with high yields, and their ease of separation and recovery is essential for large-scale green synthesis. The micro- and nanoscale nature of these catalysts ensures excellent contact between reactants and the catalyst, akin to homogeneous catalysis, while their insolubility in solvents facilitates easy separation, similar to heterogeneous catalysis. Micro- and nanoscale catalysts have proven effective in enhancing various catalytic reactions to align with the principles of green chemistry for chemical conversion. Their small size and large surface area not only increase reaction rates but also provide a bridge between homogeneous and heterogeneous catalysis [43]. As the demand for sustainable and cost-effective catalysts continues to grow, the field of micro- and nano-catalysis gains increasing importance in both academic and industrial settings. While micro/nanoscale supports generally minimize substrate diffusion issues compared to porous supports, fully loaded biocatalysts may still face minor diffusion limitations, even with small substrates. This limitation can occur if the enzyme is not properly oriented toward the reaction medium, resulting in the substrate having to diffuse between closely packed enzyme molecules, thereby increasing the tortuosity of the substrate’s path to the active site. However, when enzymes are correctly oriented with their active sites facing the reaction medium, this issue is mitigated. Crucially, all immobilized enzyme molecules on non-porous supports can access any substrate, regardless of size, which is a distinct advantage over porous supports [21,25]. Chitosan-based micro- and nanostructured particles, in particular, are valued for their robust physicochemical and biological properties, making them an environmentally friendly option for various bioproducts and applications, including enzyme immobilization, tissue engineering, food packaging, diagnostics, biosensing, pharmaceuticals, and wastewater treatment [44]. Several successful examples of protease immobilization on chitosan micro- and nanoparticles already exist [45,46]. Given the ongoing investigation into micro- or nano-biocatalysts with high medical and industrial applicability—such as enhanced enzymatic stability, operational efficiency, processability, and reusability—this work aims to develop biocatalysts based on cysteine proteases in combination with chitosan and carboxymethyl chitosan micro- and nanoparticles.

## 2. Materials and Methods

### 2.1. Materials

Cysteine proteases, specifically ficin (F4165), papain (P4762), and bromelain (B4882), were obtained from Sigma-Aldrich (Munich, Germany) and used in this research without further purification. Azocasein, also purchased from Sigma-Aldrich, was used as the hydrolysis substrate in the catalytic activity assays. Chitosan samples with molecular weights of approximately 200 kDa and 350 kDa and degrees of deacetylation ranging from 0.73 to 0.85 were obtained from Bioprogress (Shchelkovo, Russia). Chloroacetic acid (synthesis grade), provided by Sigma-Aldrich (Munich, Germany), as well as sodium hydroxide, ascorbic acid, and isopropanol, all of analytical grade, were purchased from Vekton-Center (Saint-Petersburg, Russia) and used in the synthesis of carboxymethyl chitosan.

### 2.2. Synthesis of Carboxymethyl Chitosan

Carboxymethyl chitosan was synthesized according to the following protocol: 3.0 g of chitosan was dispersed in 65 mL of isopropyl alcohol, followed by the dropwise addition of an aqueous NaOH solution over 15 min (NaOH: repeating unit = 13:1 mol/mol). Subsequently, a 15% *w*/*v* solution of chloroacetic acid in isopropyl alcohol (CH_2_ClCOOH: repeating unit = 7:1 mol/mol) was added dropwise to the reaction mixture, which was stirred for 12 h at room temperature. The resulting precipitate was filtered, suspended, washed with methanol, and dried under a vacuum at 55 ± 2 °C until a constant weight was achieved [47]. The product yield ranged from 79 to 92%. Degrees of substitution, calculated from FTIR data (Figure S2), were determined by analyzing the ratio of areas under the absorption bands at ν_s_COO^−^ (~1454–1422 cm^−1^) and νC=O (~1649–1657 cm^−1^), yielding values of 0.54 and 0.78 for carboxymethyl chitosan with molecular weights of 350 kDa and 200 kDa, respectively. The increased degree of substitution for 200 kDa of chitosan likely results from its powdered form in contrast to the flake form of the higher molecular weight sample. Given the heterogeneous nature of carboxymethylation, the form and particle size of chitosan significantly influenced the properties of the final products.

### 2.3. Obtaining Micro- or Nanoparticles of Chitosan and Carboxymethyl Chitosan

Three hundred milligrams of chitosan or carboxymethyl chitosan were dissolved in 100 mL of 0.3% acetic acid with mechanical stirring. When preparing particles in the presence of ascorbic acid, 50 mg of ascorbic acid was also added. A 3% NaOH solution was then added at a rate of 5 mL/min with continuous stirring until a white precipitate formed and the pH exceeded 11. The solution was filtered through a 0.45 μm pore size filter. The filtrate was washed with distilled water until the pH was neutral and then subjected to ultrasound treatment using a Qsonica Sonicator (Qsonica LLC., Newtown, CT, USA) for 5 min at 20 kHz for microparticles and for 10 min at 40 kHz for nanoparticles. The sizes of the micro- or nanoparticles were measured using a Nano Zetasizer ZS (Malvern Panalytical B.V., Almelo, The Netherlands). Backscattered light from a 4 mW He/Ne laser (632.8 nm) was collected at a scattering angle of 173°.

### 2.4. Preparation of Complexes of Cysteine Proteases with Micro- or Nanoparticles of Chitosan and Carboxymethyl Chitosan

Solutions of ficin (2 mg/mL in 50 mM glycine buffer, pH 10.0), papain (2 mg/mL in 50 mM glycine buffer, pH 9.0), and bromelain (2 mg/mL in 50 mM Tris-glycine buffer, pH 9.0) were mixed in equal volumes with a 0.3% *w*/*v* suspension of chitosan or carboxymethyl chitosan micro- or nanoparticles and incubated at room temperature for 2 h.

The stability of the cysteine proteases in complexes with micro- or nanoparticles was assessed at 37 °C over a period of 7 days. Catalytic activity was measured at various time intervals (0, 1, 4, 8, 24, 48, 72, 96, 120, 144, and 168 h).

### 2.5. Protein Content Assay

The protein content in the enzyme complexes with nano- or microparticles was determined using the Lowry method [48].

### 2.6. Proteolytic Activity Assay

The proteolytic activity of the complexed enzymes was assessed using the azocasein substrate [49]. The unit of catalytic activity was defined as the amount of enzyme required to hydrolyze 1 µM of the substrate in 1 min under the experimental conditions.

### 2.7. Statistical Analysis

All the experimental studies were carried out with at least 8 repetitions. Statistical processing of the results was carried out using the Stadia 8.0 Professional software package (http://protein.bio.msu.ru/~akula/Podr2~1.htm, accessed on 14 June 2024). The statistical significance of differences between the control and experimental values was determined using Student’s *t*-test (at *p* < 0.05) since all indicators were characterized by a normal distribution.

### 2.8. Molecular Docking Method

The preparation of the structures for ficin (PDB ID: 4YYW, https://www.rcsb.org/structure/4YYW, accessed on 14 June 2024), papain (PDB ID: 9PAP, https://www.rcsb.org/structure/9PAP, accessed on 14 June 2024), and bromelain (PDB ID: 1W0Q, https://www.rcsb.org/structure/1W0Q, accessed on 14 June 2024) for docking followed the standard procedure for AutoDock Vina 1.1.2 (https://sourceforge.net/projects/autodock-vina-1-1-2-64-bit/, accessed on 14 June 2024), as outlined by the software authors. The solvent, buffer, and ligand atoms and coordinates were removed from the input PDB files. The center of the molecule and the box parameters were manually set to ensure that the protease molecules were fully within the computational space.

The model of the 3D structure of chitosan and carboxymetyl chitosan was originally created using the molecular modeling software HyperChem 8.0 (https://hyperchem.software.informer.com, accessed on 14 June 2024), and subsequently, geometry was optimized by quantum-chemical calculations in PM3, which was implemented in the MOPAC 7.1 package (http://openmopac.net/, accessed on 14 June 2024). In the docking calculations, the ligand was allowed maximum conformational freedom, permitting the rotation of functional groups around all single bonds. The arrangement of charges on the polysaccharides molecule and its protonation/deprotonation were automatically handled using the MGLTools 1.5.6 package (https://ccsb.scripps.edu/mgltools/1-5-6, accessed on 14 June 2024).

## 3. Results and Discussions

### 3.1. Determination of the Size and Zeta Potential of Micro- or Nanoparticles of Medium- and High-Molecular-Weight Chitosan and Carboxymethyl Chitosan

**Figure 1:**
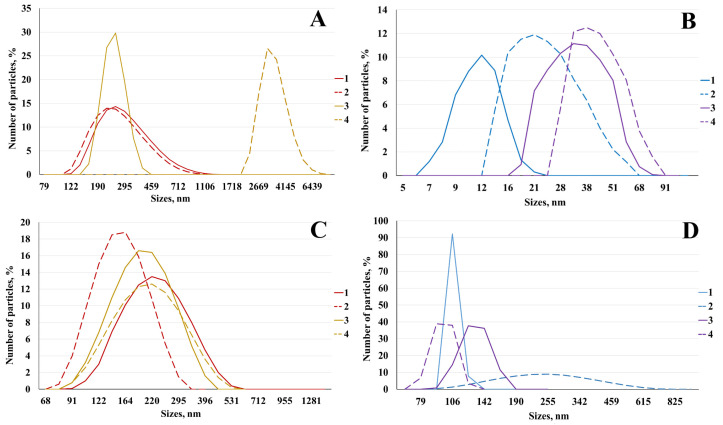
Distribution of microparticles (**A**) and nanoparticles (**B**) of chitosan and microparticles (**C**) and nanoparticles (**D**) of carboxymethyl chitosan by size: 1–200 kDa; 2–200 kDa with ascorbic acid; and 3–350 kDa; 4–350 kDa with ascorbic acid.

The size and zeta potential of micro- or nanoparticles synthesized from chitosan with molecular weights of approximately 200 and 350 kDa, prepared with and without ascorbic acid, are presented in Table 1 and Figure 1.

### 3.2. Proteolytic Activity and Stability of Ficin in Complexes with Micro- or Nanoparticles of Chitosan and Carboxymethyl Chitosan

**Figure 2:**
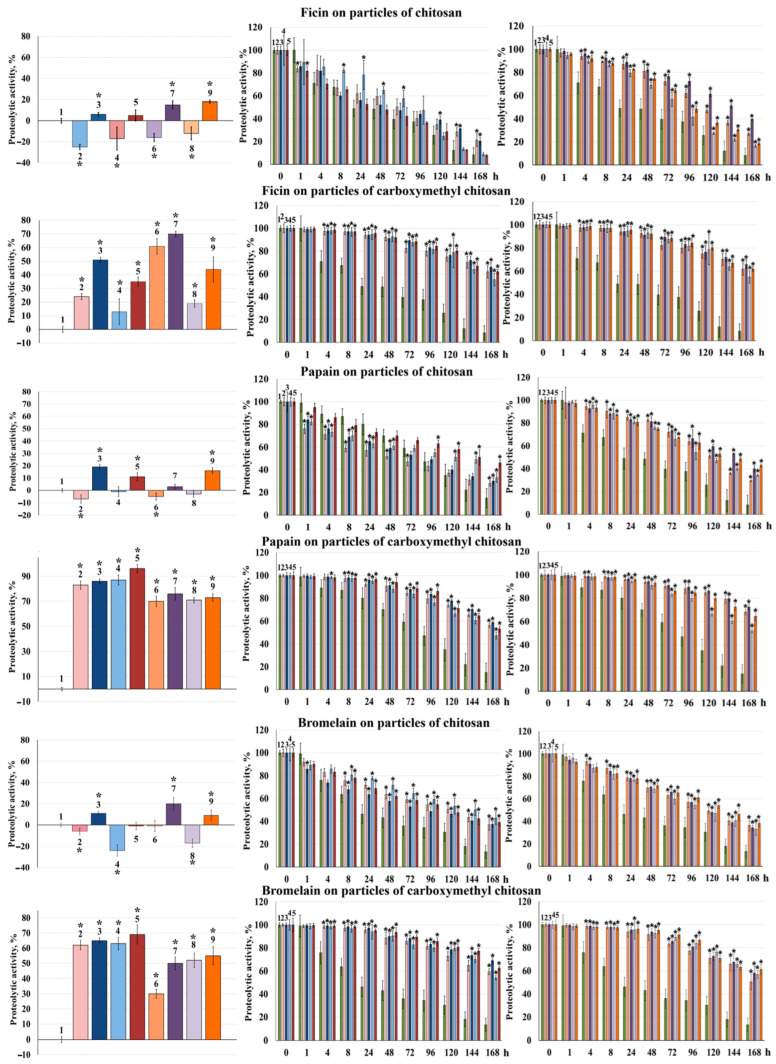
Catalytic (proteolytic) activity (**left**) and residual catalytic activity (**center**, **right**) of ficin, papain, and bromelain: free (1); in a complex with Ch200Mp/CMCh200Mp (2); Ch200MpAsc/CMCh200MpAsc (3); Ch350Mp/CMCh350Mp (4); Ch350MpAsc/CMCh350MpAsc (5); Ch200Np/CMCh200Np (6); Ch200NpAsc/CMCh200NpAsc (7); Ch350Np/CMCh350Np (8); and Ch350NpAsc/CMCh350NpAsc (9). The activity of free enzymes under optimum hydrolysis conditions (**left**) and the activity of the samples without their pre-incubation and under optimal hydrolysis conditions (**center**, **right**) were taken as 100%. * indicates statistical differences.

In complexes with Ch200Mp and Ch350Mp, ficin retained 75% and 82% of its enzymatic activity, respectively, compared to the free enzyme. Ficin activity increased by 5–6% in complexes with Ch200MpAsc and Ch350MpAsc. In complexes with Ch200Np and Ch350Np, ficin retained 83% and 87% of its activity, respectively. When ficin was complexed with Ch200NpAsc and Ch350NpAsc, its catalytic activity increased by 15% and 18%, respectively (Figure 2). The activity of ficin complexes with chitosan nanoparticles of ~200 and ~350 kDa molecular weight was higher than that of microparticle complexes by 8% and 5%, respectively, when prepared without ascorbic acid and by 9% and 13%, respectively, when prepared with ascorbic acid.

When ficin was complexed with CMCh200Mp and CMCh350Mp, the biocatalysts exhibited a 24% and 13% increase in activity, respectively, compared to the free enzyme. Ficin activity increased by 51% when complexed with CMCh200MpAsc and by 35% with CMCh350MpAsc. In complexes with CMCh200Np and CMCh350Np, the biocatalysts exhibited a 61% and 19% increase in activity, respectively, compared to the free enzyme. Ficin’s catalytic capacity increased by 70% with CMCh200NpAsc and by 44% with CMCh350NpAsc (Figure 2). The activity of ficin complexes with carboxymethyl chitosan nanoparticles (200 and 350 kDa) was higher than that of microparticle complexes by 37% and 6%, respectively, when prepared without ascorbic acid, and by 19% and 9%, respectively, when prepared with ascorbic acid.

The complexation with micro- or nanoparticles of chitosan and carboxymethyl chitosan increased the stability of ficin during incubation at 37 °C in a 0.05 M Tris-HCl buffer (pH 7.5), except for the complexes with Ch350Mp and Ch350MpAsc, which did not result in increased enzyme stability (Figure 2, Table 2).

### 3.3. Proteolytic Activity and Stability of Papain in Complexes with Micro- or Nanoparticles of Chitosan and Carboxymethyl Chitosan

When papain was complexed with Ch200Mp and Ch350Mp, the biocatalysts retained 93% and 99% of enzyme activity, respectively, compared to the free enzyme. Papain activity increased by 19% upon its interaction with Ch200MpAsc and by 11% in complexes with Ch350MpAsc. In complexes with Ch200Np and Ch350Np, the biocatalysts retained over 95% of enzyme activity, with papain’s catalytic activity increasing by 16% when complexed with Ch350NpAsc (Figure 2).

The complexation of papain with CMCh200Mp and CMCh350Mp led to an 83% and 87% increase in activity, respectively, compared to the free enzyme. Papain activity increased by 86% in complexes with CMCh200MpAsc and by 96% with CMCh350MpAsc (Figure 2).

When complexed with Ch200Np and Ch350Np, the biocatalysts exhibited an approximately 70% increase in activity compared to the free enzyme. Complexation with nanoparticles synthesized in the presence of ascorbic acid further enhanced papain’s catalytic activity by 76% for Ch200NpAsc and 73% for Ch350NpAsc. The activity of papain complexes with Ch200Mp and Ch350Mp was 13% and 16% higher, respectively, than that of the corresponding nanoparticle complexes. Similarly, the activity of papain complexes with Ch200MpAsc and Ch350MpAsc was 12% and 23% higher, respectively, than that of the corresponding nanoparticle complexes (Figure 2).

Complexation with micro- or nanoparticles of chitosan and carboxymethyl chitosan increased the stability of papain during incubation at 37 °C in a 0.05 M Tris-HCl buffer (pH 7.5) (Figure 2, Table 2).

### 3.4. Proteolytic Activity and Stability of Bromelain in Complexes with Micro- or Nanoparticles of Chitosan and Carboxymethyl Chitosan

When bromelain was complexed with Ch200Mp and Ch350Mp, the biocatalysts retained 94% and 76% of the free enzyme’s activity, respectively. The activity of bromelain complexes increased by 11% upon their interaction with Ch200MpAsc and remained unchanged in complexes with Ch350MpAsc. When complexed with Ch200Np and Ch350Np, the biocatalysts retained 99% and 83% of the free enzyme’s activity, respectively. Complexation with Ch200NpAsc and Ch350NpAsc enhanced bromelain’s catalytic activity by 21% and 9%, respectively (Figure 2).

In complexes with CMCh200Mp and CMCh350Mp, bromelain’s activity was 63% and 52% higher than that of the free enzyme, respectively. Bromelain activity increased by 69% in complexes with CMCh200MpAsc and by 55% with CMCh350MpAsc. When complexed with CMCh200Np and CMCh350Np, the biocatalysts exhibited increases in activity of 62% and 30%, respectively, compared to the free enzyme. Complexation with nanoparticles synthesized in the presence of ascorbic acid further enhanced bromelain’s catalytic activity by 65% for CMCh200NpAsc and by 50% for CMCh350NpAsc. Notably, the activity of bromelain complexes with CMCh350Mp was 22% higher than that of the corresponding nanoparticle complexes (Figure 2).

Complexation with micro- or nanoparticles of chitosan and carboxymethyl chitosan enhanced bromelain stability during incubation at 37 °C in a 0.05 M Tris-HCl buffer (pH 7.5). Additionally, the stabilizing effect of chitosan microparticles on bromelain was significantly more pronounced than on ficin and papain (Figure 2, Table 2).

### 3.5. Molecular Docking

To investigate the influence of a complex formation with micro- or nanoparticles of chitosan and carboxymethyl chitosan on the activity and stability of cysteine proteases, a molecular docking study was conducted.

The study revealed that, during the formation of the ficin–chitosan complex, five hydrogen bonds were formed, whereas four hydrogen bonds were formed in the ficin–carboxymethyl chitosan complex. Asp161 is the only residue involved in hydrogen bonding with both types of polysaccharides (Table 3).

In the formation of the ficin–chitosan–ascorbic acid and ficin–carboxymethyl chitosan–ascorbic acid complexes, a total of 17 hydrogen bonds were formed, with several amino acid residues (Gly20, Glu121, Glu145, Asp161, Asn199, Val200, Glu202, Pro203) participating in bonding with both polysaccharides (Table 3).

**Figure 3:**
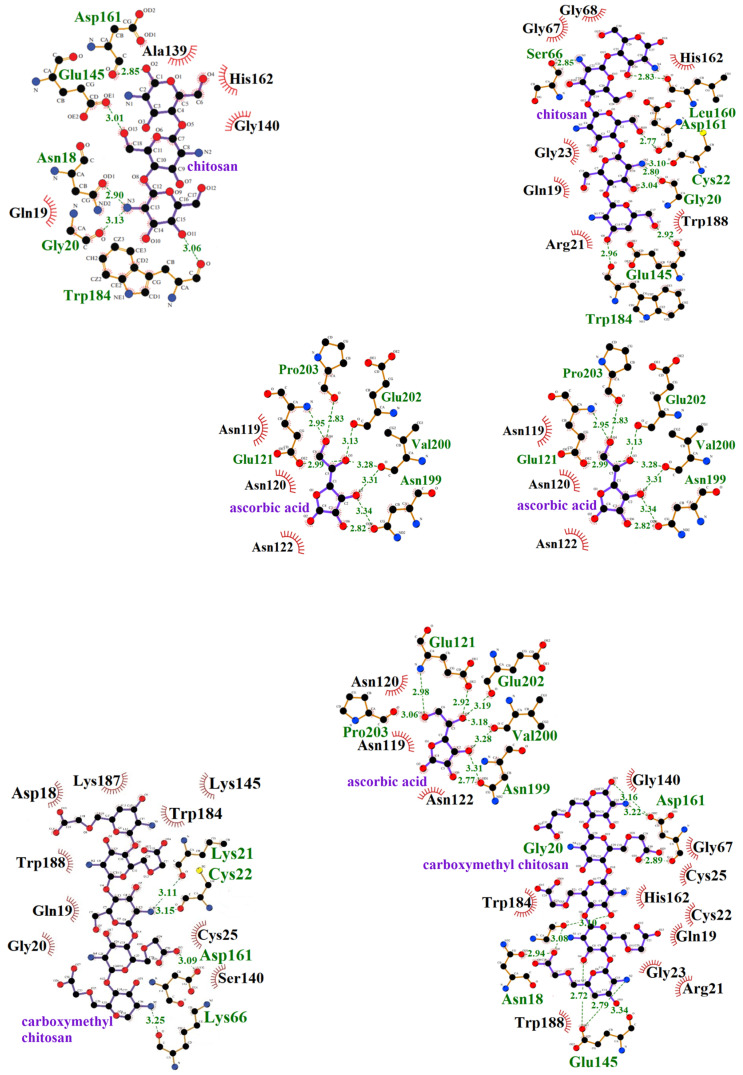
Bonds and interactions in complexes of ficin with chitosan and carboxymethyl chitosan with and without ascorbic acid: dashed lines indicate hydrogen bonds, and their lengths are given in Å. Red arcs indicate the presence of weak physical interactions (electrostatic, hydrophobic, and van der Waals) between the amino acid residues of the enzyme and the carrier/ligand. C-atoms are black, O-atoms are red, N-atoms are blue.

The same interactions that occurred between ficin and chitosan and between ficin and ascorbic acid drove the formation of the ficin–chitosan–ascorbic acid complex. Additionally, new amino acid residues—Arg21, Cys22, Gly23, Ser66, Gly67, Gly68, and Trp188—contribute to the ficin–chitosan–ascorbic acid ternary complex (Table 3). Similarly, interactions between ficin and carboxymethyl chitosan, as well as between ficin and ascorbic acid, contribute to the formation of the ficin–carboxymethyl chitosan–ascorbic acid complex (Figure 3). New amino acid residues—Asn18, Gly20, Arg21, Cys22, Gly23, Gly67, Gly68, Glu145, and His162—also participate in this ternary complex (Figure 3, Table 3).

In the ficin–chitosan, ficin–chitosan–ascorbic acid, and ficin–carboxymethyl chitosan–ascorbic acid complexes, hydrophobic interactions involve His162, an active site residue of the enzyme. Additionally, in the ficin–carboxymethyl chitosan and ficin–carboxymethyl chitosan–ascorbic acid complexes, the active site residue Cys25 also participates in the interactions (Table 3).

**Figure 4:**
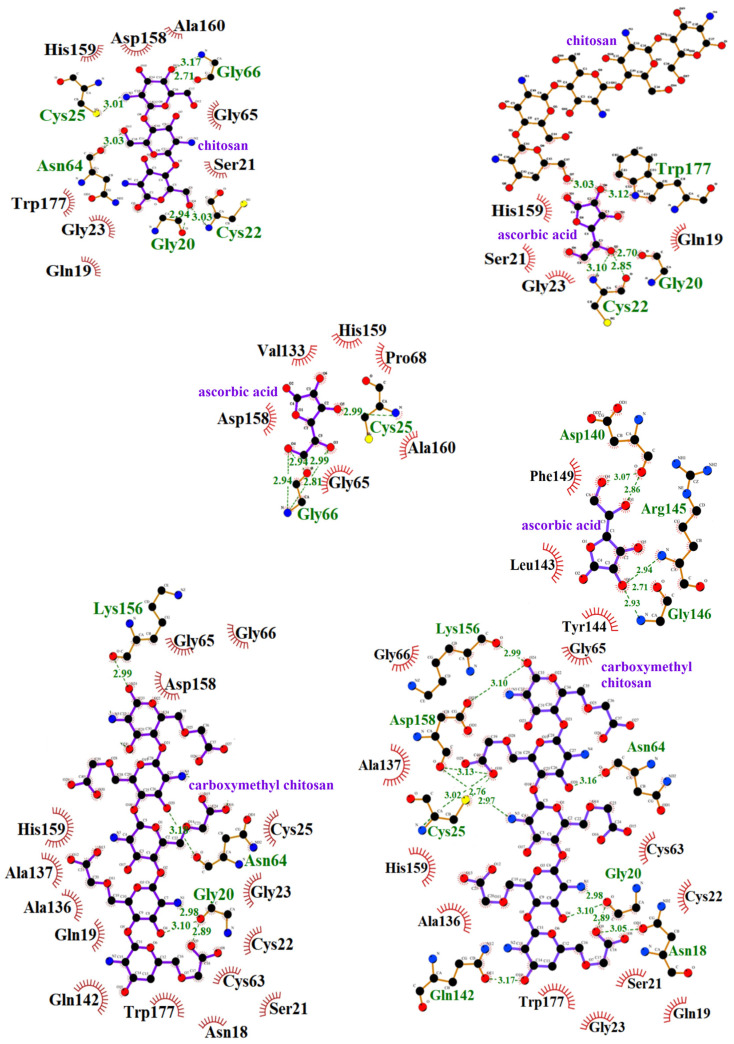
Bonds and interactions in complexes of papain with chitosan and carboxymethyl chitosan with and without ascorbic acid: dashed lines indicate hydrogen bonds, and their lengths are given in Å. Red arcs indicate the presence of weak physical interactions (electrostatic, hydrophobic, and van der Waals) between the amino acid residues of the enzyme and the carrier/ligand. C-atoms are black, O-atoms are red, N-atoms are blue.

In the formation of the bromelain–chitosan complex, eight hydrogen bonds are established, while adsorption onto carboxymethyl chitosan leads to the formation of ten hydrogen bonds. The amino acid residues Asn19, Glu51, Lys179, and Trp180 are involved in hydrogen bonding with both types of polysaccharides (Table 3, Figure 4). The bromelain–chitosan–ascorbic acid complex forms 12 hydrogen bonds, whereas 21 hydrogen bonds are formed in the bromelain–carboxymethyl chitosan–ascorbic acid complex. Several amino acid residues (Lys18, Glu36, Thr161, Ile163, Gln167, Lys179, Ile186) contribute to hydrogen bonding with both polysaccharides (Table 3). The interactions that contribute to the bromelain–chitosan–ascorbic acid complex are also present between bromelain and chitosan (Figure 4) and bromelain and ascorbic acid. The Thr15 residue forms hydrogen bonds with both ascorbic acid and chitosan, playing a role in the formation of the bromelain–chitosan–ascorbic acid ternary complex. Additional residues—Asp7, Arg9, Val14, Ser16, Gln141, Lys144, Thr161, Ala162, Gly164, Gln167, Trp180, Tyr185, Ile186, Arg187—participate in forming this ternary complex (Figure 3, Table 3). In the bromelain–carboxymethyl chitosan–ascorbic acid complex, residues Val17, Ala33, and Glu36 interact with both ascorbic acid (Figure 4) and carboxymethyl chitosan, contributing to the ternary complex. Additional residues, including Asp7, Arg9, Ala136, Thr161, Ala162, Ile163, Gly164, Gln167, Gly184, Ile186, and Arg187, also participate in this ternary complex (Table 3). Within the bromelain–chitosan complex, a single hydrogen bond is formed involving His158, which is an active site residue. Adsorption onto the carboxymethyl chitosan matrix results in His158 forming two salt bridges with the carrier matrix.

**Figure 5:**
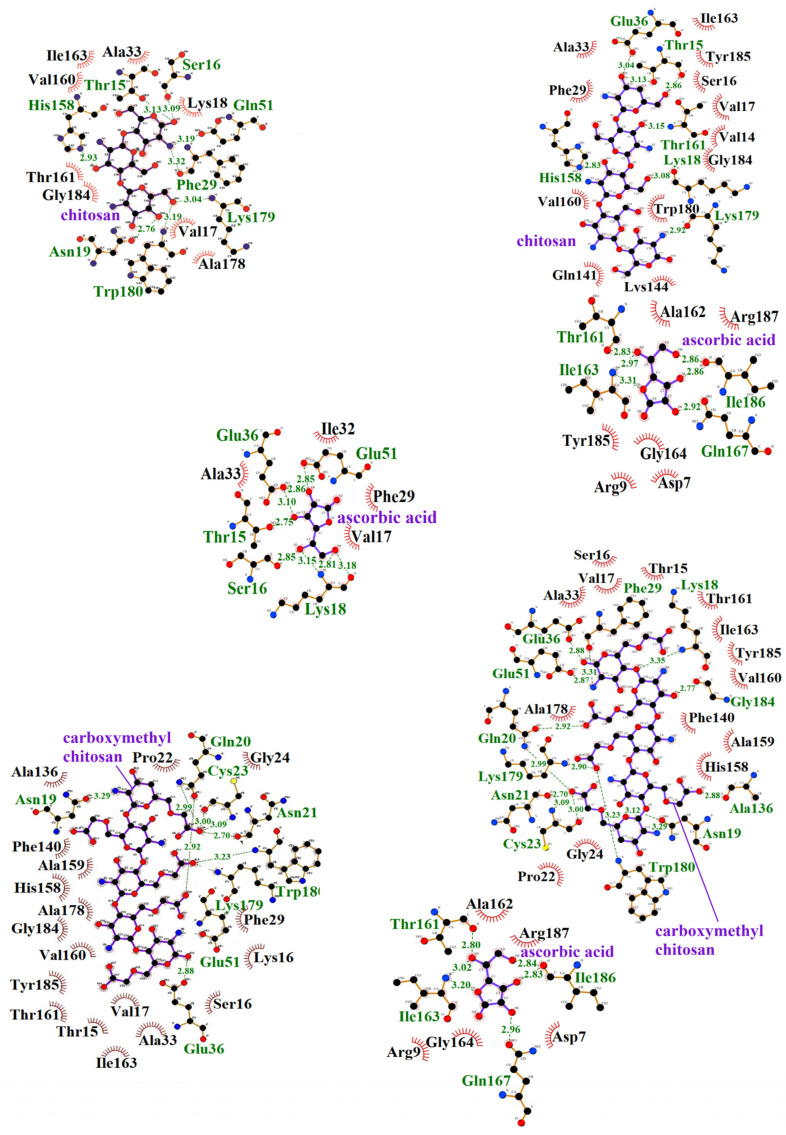
Bonds and interactions in bromelain complexes with chitosan and carboxymethyl chitosan with and without ascorbic acid: dashed lines indicate hydrogen bonds, and their lengths are given in Å. Red arcs indicate the presence of weak physical interactions (electrostatic, hydrophobic, and van der Waals) between the amino acid residues of the enzyme and the carrier/ligand. C-atoms are black, O-atoms are red, N-atoms are blue.

In the formation of the papain–chitosan complex, six hydrogen bonds are formed, while five are created when papain is adsorbed onto carboxymethyl chitosan. The amino acid residues Gly20 and Asn64 contribute to hydrogen bonding with both polysaccharides (Table 3). The papain–chitosan–ascorbic acid complex forms five hydrogen bonds, while the papain–carboxymethyl chitosan–ascorbic acid complex forms 17 hydrogen bonds. Only the Gly20 residue is involved in hydrogen bonding with both polysaccharides (Table 3). Interactions between papain and chitosan cause the formation of the papain–chitosan–ascorbic acid complex. Gly20 and Cys22 form hydrogen bonds with chitosan and contribute to the ternary papain–chitosan–ascorbic acid complex. A unique feature of this complex is the hydrogen bond formed between ascorbic acid and chitosan. Similarly, the interactions between papain and carboxymethyl chitosan, as well as those between papain and ascorbic acid, contribute to the formation of the papain–carboxymethyl chitosan–ascorbic acid complex. Additional amino acid residues—Asn18, Asp140, Gln142, Leu143, Tyr144, Arg145, Gly146, and Phe149—participate in forming this complex (Figure 5, Table 3).

In both the papain–chitosan and papain–carboxymethyl chitosan–ascorbic acid complexes, a hydrogen bond is formed with Cys25, and hydrophobic interactions involving His159 are observed. Both residues are part of papain’s active site.

### 3.6. Hybrid Catalysts of Cysteine Proteases Enhanced by Chitosan and Carboxymethyl Chitosan Micro- or Nanoparticles Offer Distinct Advantages over Other Cysteine Protease Samples Immobilized on Chitosan and Its Derivatives

Attempts to obtain ficin, papain, and bromelain immobilized on chitosan or its derivatives have been made repeatedly [50]; however, several unresolved issues in this area remain. In particular, most studies have focused on chitosan macroparticles [51,52], the disadvantages of which were outlined in the introduction. Additionally, the activity of ficin, papain, and bromelain is often significantly decreased when immobilized on chitosan macroparticles [5,6,49,53], and these samples are soluble and functionally active only in acidic media with pH < 6.5. To expand the solubility range of the samples, chemically modified chitosan derivatives have been utilized as carriers, including 2-(4-acetamido-2-sulfanilamide) chitosan, carboxymethyl chitosan, and *N*-(2-hydroxy)propyl-3-trimethylammonium chitosan, which have been previously proposed as matrices for protease immobilization [9,54,55].

A new direction involves the production of chitosan membranes containing 2.5% and 5.0% papain, which significantly increased enzymatic activity to 0.87 ± 0.12 U/mg and 1.59 ± 0.10 U/mg, respectively, compared to the free enzyme (0.0042 ± 0.001 U/mg). However, increasing the papain concentration to 10% or more led to phase separation in the membrane, while the presence of papain reduced the hydrophobicity of the membrane surface and impaired its swelling ability [56].

In all the aforementioned studies, additional substances were required to prevent the oxidation of the thiol group in the active site of cysteine proteases. In this study, ascorbic acid was employed in the composition of papain complexes with polysaccharides for this purpose. Ascorbic acid is a natural, water-soluble antioxidant, primarily acting to terminate chain radical processes through electron transfer [57]. Additionally, ascorbic acid molecules can serve as absorbers of molecular oxygen [58]. Furthermore, ascorbic acid is known for its antimicrobial, bactericidal, and antiviral properties and is often used as a “capping” agent to prevent the aggregation of nanoparticles [59]. Given its low toxicity and widespread application in the food and pharmaceutical industries, the inclusion of ascorbic acid in immobilized papain preparations could facilitate the creation of stable and highly active micro- and nano-sized biocatalysts with a broad spectrum of action.

## 4. Conclusions

Enzymes are highly effective biocatalysts; however, despite their numerous advantageous properties, they often exhibit instability and are susceptible to inhibition by various compounds. This issue can be addressed by immobilizing enzymes onto micro- and nanocarriers. When developing micro- and nanoscale biocomplexes, it is crucial to consider the affinity between the functional groups of the enzyme and the carrier. Molecular docking studies have indicated that chitosan and carboxymethyl chitosan are promising carriers for the immobilization of proteolytic enzymes, which is a finding that has been experimentally validated. In experiments assessing the activity of cysteine proteases, it was observed that the activity of ficin complexes with carboxymethyl chitosan microparticles was 1.7 times higher than that with Ch200Mp and 1.4 times higher than that with Ch350Mp. The use of carboxymethyl chitosan nanoparticles resulted in ficin activity that was 1.9 times higher than with Ch200Np and 1.4 times higher than with Ch350Np. Similarly, the activity of papain complexes with carboxymethyl chitosan microparticles was twice as high as that with Ch200Mp, 1.9 times higher for Ch350Mp, and 1.8 times higher for both types of chitosan nanoparticles. For bromelain, complexes with carboxymethyl chitosan microparticles demonstrated 1.7 times higher activity compared to Ch200Mp, 2 times higher for Ch350Mp, and 1.6 times higher for both types of chitosan nanoparticles.

These resulting complexes have potential applications in the food industry for food production, in medicine and pharmaceuticals as therapeutic agents, and in various industrial and biotechnological processes.

## Figures and Tables

**Table 1:** Parameters of micro- or nanoparticles.

Sample	Medium Size, nm	Size Range, nm	Median Zeta Potential Value, mV	Zeta Potential Range, mV
**Chitosan microparticles**				
molecular weight of ~200 kDa (Ch200Mp)	220	122–1281	0	0
molecular weight of ~200 kDa + ascorbic acid (Ch200MpAsc)	190	106–1106	0	0
molecular weight of ~350 kDa (Ch350Mp)	220	142–459	0	0
molecular weight of ~350 kDa + ascorbic acid (Ch350MpAsc)	2670	1990–7456	0	0
**Chitosan** **nanoparticles**				
molecular weight of ~200 kDa (Ch200Np)	12	6–24	0	0
molecular weight of ~200 kDa + ascorbic acid (Ch200NpAsc)	21	12–68	0	0
molecular weight of ~350 kDa (Ch350Np)	33	16–79	0	0
molecular weight of ~350 kDa + ascorbic acids (Ch350NpAsc)	38	24–91	0	0
**Carboxymethyl chitosan microparticles**				
molecular weight of ~200 kDa (CMCh200Mp)	220	91–615	0	0
molecular weight of ~200 kDa + ascorbic acid (CMCh200MpAsc)	164	68–342	0	0
molecular weight of ~350 kDa (CMCh350Mp)	190	79–459	0	0
molecular weight of ~350 kDa + ascorbic acid (CMCh350MpAsc)	220	79–615	0	0
**Carboxymethyl chitosan nanoparticles**				
molecular weight of ~200 kDa (CMCh200Np)	106	91–142	0	0
molecular weight of ~200 kDa + ascorbic acid (CMCh200NpAsc)	255	79–825	0	0
molecular weight of ~350 kDa (CMCh350Np)	122	79–190	0	0
molecular weight of ~350 kDa + ascorbic acid (CMCh350NpAsc)	91	68–142	0	0

**Table 2:** Minimal incubation time increasing enzyme stability for incubation at 37 °C in a 0.05 M Tris-HCl buffer (pH 7.5).

Type of Particles	Minimal Incubation Time Increasing Enzyme Stability in Hours		
	Ficin Complexes	Papain Complexes	Bromelain Complexes
Ch200Mp	144	168	8
Ch200MpAsc	120	168	8
Ch350Mp	complexation did not lead to increased enzyme stability	120	8
Ch350MpAsc	complexation did not lead to increased enzyme stability	96	8
Ch200Np	4	4	4
Ch200NpAsc	4	4	4
Ch350Np	4	4	8
Ch350NpAsc	4	4	8
CMCh200Mp	4	8	4
CMCh200MpAsc	4	8	4
CMCh350Mp	4	4	4
CMCh350MpAsc	4	8	4
CMCh200Np	4	4	4
CMCh200NpAsc	4	4	4
CMCh350Np	4	8	4
CMCh350NpAsc	4	8	4

**Table 3:** Binding sites in the researched complexes.

Formed Complexes	Affinity, Kcal/Mol	Amino Acid Residues That Form the Following	
		Hydrogen Bonds and Bond Length, Å	Other Types of Interactions
**Ficin and ascorbic acid**	−5.2	Glu121, 2.95 and 2.99; Asn199, 2.82 and 3.34; Val200, 3.28 and 3.31; Glu202, 3.13; Pro203, 2.83	Asn119; Asn120; Asn122
**Ficin and chitosan**	−7.7	Asn18, 2.90; Gly20, 3.13; Glu145, 3.01; Asp161, 2.85; Trp184, 3.06	Gln19; Ala139, Gly140; His162
**Ficin, chitosan, and ascorbic acid**	−5.1	Gly20, 2.80 and 3.04; Cys22, 3.10; Ser66, 2.85; Glu121, 2.95 and 2.99; Glu145, 2.92; Leu160, 2.83; Asp161, 2.77; Trp184, 2.96; Asn199, 2.82 and 3.34; Val200, 3.28 and 3.31; Glu202, 3.13; Pro203, 2.83	Gln19; Arg21; Gly23; Gly67; Gly68; Asn119; Asn120; Asn122; His162; Trp188
**Ficin and carboxymethyl chitosan**	−6.1	Lys21, 3.11; Cys22, 3.15; Lys66, 3.25; Asp161, 3.09	Asp18; Gln19; Gly20; Cys25; Gly140; Lys145; Trp184; Lys187; Trp188
**Ficin, carboxymethyl chitosan, and ascorbic acid**	−5.1	Asn18, 2.94; Gly20, 3.08 and 3.10; Glu121, 2.92 and 2.98; Glu145, 2.72, 2.79 and 3.34; Asp161, 2.89, 3.16 and 3.22 Asn199, 2.77 and 3.31; Val200, 3.18 and 3.28; Glu202, 3.19; Pro203, 3.06	Gln19; Arg21; Cys22; Gly23; Cys25; Gly67; Asn119; Asn120; Asn122; Gly140; His162; Trp184; Trp188
**Papain and ascorbic acid**	−4.8	Cys25, 2.99; Gly66, 2.94, 2.94, 2.81 and 2.99	Gly65; Pro68; Val133; Asp158; His159; Ala160
**Papain and chitosan**	−7.7	Gly20, 2.94; Cys22, 3.03; Cys25, 3.01; Asn64, 3.03; Gly66, 2.71 and 3.17	Gln19; Ser21; Gly23; Gly65; Asp158; His159; Ala160; Trp177
**Papain, chitosan and ascorbic acid**	−5.0	Gly20, 2.70; Cys22, 2.85 and 3.10; Trp177, 3.12; H-bond between ascorbic acid and chitosan, 3.03	Gln19; Ser21; Gly23; His159
**Papain and carboxymethyl chitosan**	−6.6	Gly20, 2.89, 2.98 and 3.10; Asn64, 3.18; Lys156, 2.99	Asn18; Gln19; Ser21; Cys22; Gly23; Cys25; Cys63; Gly65; Gly66; Ala136; Ala137; Gln142; Asp158; His159; Trp177
**Papain, carboxymethyl chitosan, and ascorbic acid**	−4.4	Asn18, 3.05; Gly20, 2.89, 2.98 and 3.10; Cys25, 2.76 and 3.02; Asn64, 3.16; Asp140, 2.86 and 3.07; Gln142, 3.17; Arg145, 2.94; Gly146, 2.71 and 2.93; Lys156, 2.99; Asp158, 2.97, 3.10 and 3.13	Gln19; Ser21; Cys22; Gly23; Cys63; Gly65; Gly66; Ala136; Ala137; Leu143; Tyr144; Phe149; His159; Trp177
**Bromelain and ascorbic acid**	−5.7	Thr15, 2.75; Ser16, 2.85; Lys18, 2.81, 3.15 and 3.18; Glu36, 2.86 and 3.10	Val17; Phe29; Ile32; Ala33
**Bromelain and chitosan**	−8.0	Thr15, 3.13; Ser16, 3.09; Asn19, 2.76; Phe29, 3.32; Gly51, 3.19; His158, 2.93; Lys179, 3.04; Trp180, 3.19	Val17; Lys18; Ala33; Val160; Thr161; Ile163; Ala178; Gly184
**Bromelain, chitosan, and ascorbic acid**	−5.7	Thr15, 2.86 and 3.13; Lys18, 3.08; Glu36, 3.04; His158, 2.83; Thr161, 2.83 and 3.15; Ile163, 2.97 and 3.31; Gln167, 2.92; Lys179, 2.92; Ile186, 2.86 and 2.86	Asp7; Arg9; Val14; Ser16; Val17; Phe29; Ala33; Gln141; Lys144; Val160; Ala162; Ile163; Gly164; Trp180; Gly184; Tyr185; Arg187
**Bromelain and carboxymethyl chitosan**	−8.4	Asn19, 3.29; Gln20, 2.92 and 2.99; Asn21, 2.70; Cys23, 3.00 and 3.09; Glu36, 2.88; Glu51, 2.87; Lys179, 2.90; Trp180, 3.23	Thr15; Ser16; Val17; Lys16; Pro22; Gly24; Phe29; Ala33; Ala136; Phe140; His158; Ala159; Val160; Thr161; Ile163; Ala178; Gly184; Tyr185
**Bromelain, carboxymethyl chitosan, and ascorbic acid**	−5.8	Lys18, 3.35; Asn19, 3.12 and 3.29; Gln20, 2.92 and 2.99; Asn21, 2.70; Cys23, 3.00 and 3.09; Phe29, 3.31; Glu36, 2.88; Glu51, 2.87; Ala136, 2.88; Thr161, 2.80; Ile163, 3.02 and 3.20; Gln167, 2.96; Lys179, 2.90; Trp180, 3.23; Gly184, 2.77; Ile186, 2.83 and 2.84	Asp7; Arg9; Thr15; Ser16; Val17; Pro22; Gly24; Ala33; Phe140; His158; Ala159; Val160; Thr161; Ala162; Ile163; Gly164; Ala178; Tyr185; Arg187

## Data Availability

The original contributions presented in the study are included in the article/Supplementary Materials, and further inquiries can be directed to the corresponding authors.

## Supplementary Materials

The following supporting information can be downloaded at https://www.mdpi.com/article/10.3390/polym16223111/s1, Figure S1: Fragments of chitosan and carboxymethyl chitosan molecules; Figure S2: FTIR spectra of chitosan and carboxymethyl chitosan.
