# Supplementary material for: Novel Hybrid Catalysts of Cysteine Proteases Enhanced by Chitosan and Carboxymethyl Chitosan Micro- and Nanoparticles
Source: Polymers (Basel). 2024 Nov 6;16(22):3111. doi: 10.3390/polym16223111 (PMC11598686; doi:10.3390/polym16223111)
Supplement: Supplementary file 1 [file polymers-16-03111-s001.zip › polymers-3283884-supplementary.pdf]

Novel Hybrid Catalysts of Cysteine Proteases Enhanced by Chitosan and Carboxymethyl Chitosan Micro- and Nanoparticles

Marina Holyavka, Yulia Redko, Svetlana Goncharova, Maria Lavlinskaya, Andrey Sorokin, Maxim Kondratyev, Valery Artyukhov

Supplementary materials

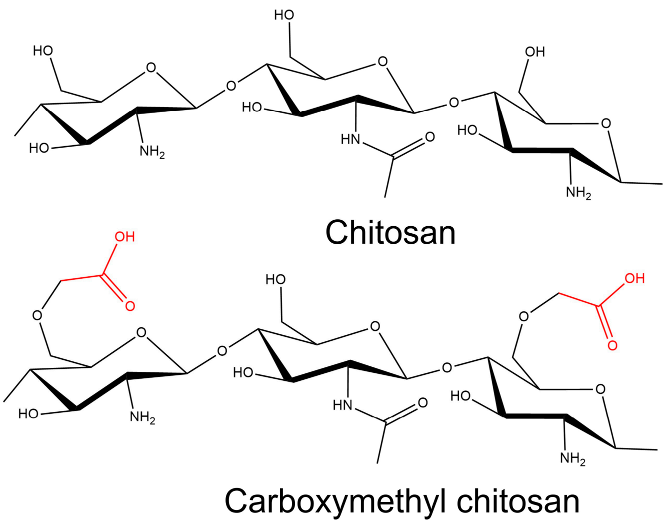

Figure S1. Fragments of chitosan and carboxymethyl chitosan molecules.

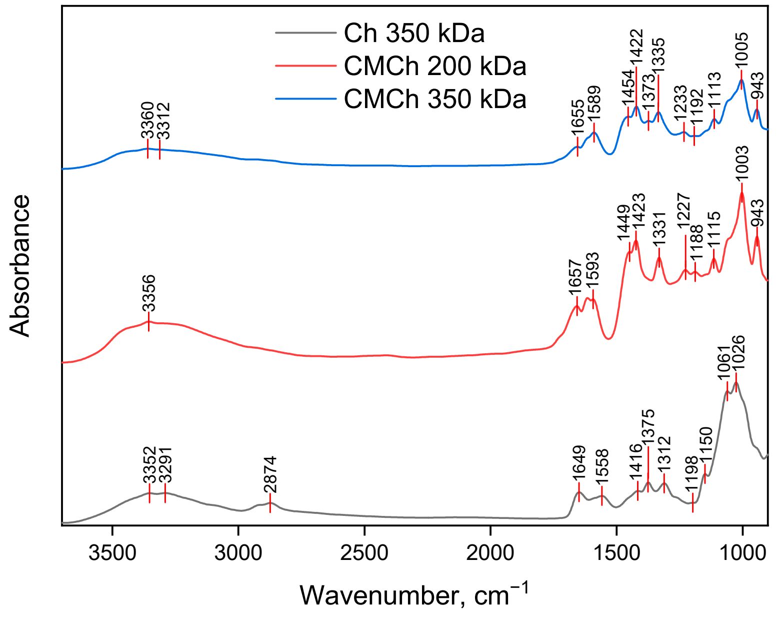

Figure S2. FTIR spectra of chitosan and carboxymethyl chitosan.
